# Prenatal THC exposure disrupts mitochondrial respiratory gene programs and delays medium spiny neuron maturation in the nucleus accumbens

**DOI:** 10.64898/2026.04.26.720961

**Published:** 2026-04-28

**Authors:** Zhong Chen, Wanqiu Chen, Yun Seok Lee, Wendell Jones, Laura Goetzl, Jennifer Dawn Thomas, Yan Dong, Charles Wang

**Affiliations:** 1 Center for Genomics, School of Medicine, Loma Linda University, Loma Linda, CA 92350, USA; 2 Department of Basic Sciences, School of Medicine, Loma Linda University, Loma Linda, CA 92350, USA; 3 IQVIA Laboratories Genomics, Durham, NC, USA; 4 Department of Obstetrics, Gynecology and Reproductive Sciences, McGovern Medical School, University of Texas Health at Houston, Houston, TX 77030, USA; 5 Center for Behavioral Teratology, San Diego State University, San Diego, CA 92120, USA; 6 Department of Psychiatry, University of Pittsburgh, Pittsburgh, PA 15260, USA

## Abstract

Prenatal cannabis exposure (PCE) is increasingly prevalent and has been associated with adverse neurodevelopment outcomes, yet its molecular impact on brain reward circuitry remains poorly defined. Here, we investigated transcriptional and epigenomic alterations in the nucleus accumbens (NAc) following prenatal Δ^9^-tetrahydrocannabinol (THC) exposure in a rat model using snRNA-seq and snATAC-seq analyses. PCE markedly suppressed the expression of genes involved in mitochondrial oxidative phosphorylation in the NAc on postnatal day 24 (P24), consistent with impaired mitochondrial respiration capacity. This mitochondrial dysfunction was accompanied by coordinated alterations in ribosomal and proteasomal pathways regulating protein homeostasis in NAc medium spiny neurons (MSNs), indicating coupled disruption of cellular metabolism and neuronal maturation. snATAC-seq analysis revealed altered chromatin accessibility at promoter regions enriched with NRF1 and YY2 binding motifs, highlighting the importance of transcriptional regulation of mitochondrial gene in MSNs after PCE. Moreover, an acute THC challenge in PCE offspring at P24 further exacerbated mitochondrial dysfunction and delayed MSN maturation. Together, these findings define a transcriptional and epigenetic framework through which PCE perturbs mitochondrial function and impairs MSN maturation in the NAc, providing mechanistic insights into how PCE may alter the development of reward circuity.

## Introduction

Cannabis is one of the most commonly used substances in pregnant women^1, 2^. A recent study has indicated a prevalence as high as 22.4% for detectable prenatal marijuana expose from umbilical cord blood sample^3^. Prenatal cannabis exposure (PCE) poses health risks to the developing fetus, as the primary psychoactive constituent Δ^9^ tetrahydrocannabinol (THC), readily crosses the placenta, leading to long-term neurodevelopmental disorders including impaired cognitive function, hyperactivity, and increased impulsivity^4–6^. Given the rising prevalence of maternal cannabis use and its deleterious effects, it is critical to investigate the impact of PCE on fetal brain development.

The nucleus accumbens (NAc) is a key brain region regulating emotional and motivational responses to both natural rewards and drugs of abuse^7–9^. Cannabis exposure has been associated with alterations in the neural architecture of core reward structures^10^. For example, animal studies have shown that cannabinoids induce structural abnormalities^11^ and altered synaptic transmission in the NAc^12^. However, it remains unclear how PCE affects the development and function of the NAc at cellular and molecular levels.

Mitochondria undergo morphological change and a metabolic shift from glycolysis to oxidative phosphorylation during differentiation in both cardiac and motor neurons^13, 14^. Similar changes, including increased mitochondrial oxygen consumption, have been observed during embryonic stem cell (ESC) differentiation^15^. Inhibition of mitochondrial respiration impairs differentiation and enhancement of stem cell pluripotency^16^, while mitochondrial oxidative activity and glucose metabolism play a key role in regulating the tempo of neuronal maturation^17^.

Biochemical and cytological studies indicate that THC disrupts mitochondrial function in the brain^18^. Several molecular mechanisms have been proposed. Initially, THC was thought to act directly on mitochondria due to their high lipophilic membranes, potentially disturbing mitochondrial membrane properties via destabilization of cardiolipin^19^. Subsequent studies suggest that THC can also regulate mitochondrial calcium levels, impair the electron transport chain, disrupt mitochondrial oxidative phosphorylation (OXPHOS) and ATP production, or alter mitochondrial biogenesis dynamics^18, 20^. Collectively, these findings suggest that THC-induced mitochondrial dysfunction may critically contribute to the adverse effect of PCE on neuronal development, particularly in the context of temporal and cell-type specific dynamics revealed by single-cell transcriptomic level.

We hypothesized that PCE disrupts the expression of nucleus-encoded mitochondrial genes in NAc neurons by altering transcriptional regulation and chromatin accessibility at single-cell level resolution, thereby impairing neuronal maturation and function. To test this, we performed snRNA-seq and snATAC-seq on NAc tissues from postnatal day 24 (P24) PCE rats, along with snRNA-seq following acute THC challenge in PCE male offspring. PCE suppressed the expression of genes involved in the mitochondrial respiratory chain and broadly impaired mitochondrial function across multiple NAc cell types, with the most pronounced effects in medium spiny neurons (MSNs). Acute THC challenge in PCE offspring further exacerbated mitochondrial dysfunction and shifted MSN transcriptomic profiles toward a less mature state, suggesting increased vulnerability of reward circuitry to subsequent cannabinoid exposure.

## Results

### Rat NAc cell type identification

We simultaneously profiled gene expression (GEX) and chromatin accessibility (ATAC-seq) from the same single nuclei isolated from the NAc, using the 10x Multiome chemistry. The nuclei were isolated from a pool of NAc tissues collected from four P24 rats derived from three dams across four groups: female control (F_Ctl), female prenatal cannabis exposure (F_PCE), male control (M_Ctl), and male prenatal cannabis exposure (M_PCE). Prenatal THC exposure was administered from embryonic day 5 (E5) to E20, spanning implantation through late gestation, a developmental window roughly corresponding to the late first trimester through term in humans.

**Fig. 1.**
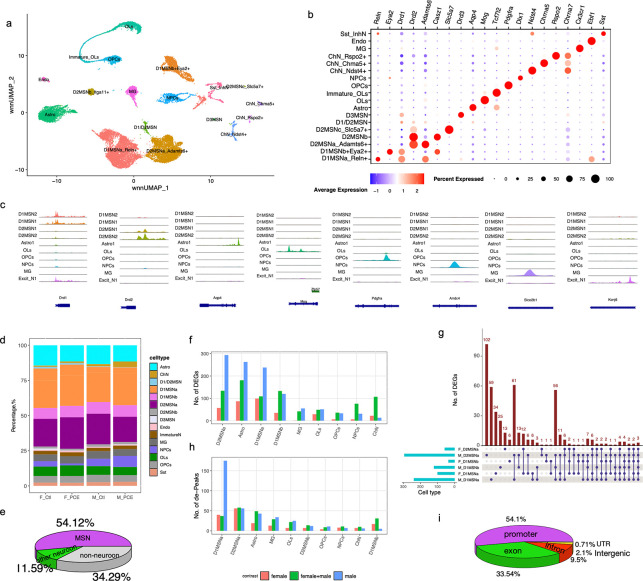
PCE induces transcriptomic and chromatin structural changes in P24 rat NAc (**a**) Unsupervised cell clustering based on wnn_UMAP of Multiome data from F_Ctl, F_PCE, M_Ctl, and M_PCE groups. (**b**) Dot Plot showing the expression of cluster-specific marker genes. (**c**) Peak coverage tracks highlighting cell type-specific ATAC-seq signals across major cell populations. The three ChN subtypes were combined to increase ATAC signal intensity. (**d**) Bar plot illustrating cell-type composition across four datasets; clusters containing fewer than 20 cells were excluded. (**e**) Pie chart showing the proportion of all MSNs among all cells combined from four datasets. (**f, h**) Bar plots showing the number of DEGs (**f**) and differential chromatin accessibility peaks (**h**) identified in each cell type. (**g**) UpSet plot illustrating shared and unique DEGs among D1MSNa, D1MSNb, and D2MSNa populations in male and female PCE rat NAc. (**i**) Pie chart showing the genomic annotation distribution of differential peaks induced by PCE.

We obtained 24,095 nuclei with matched transcriptomic and chromatin accessibility profiles (F_Ctl: 6,203; F_PCE: 6,446; M_Ctl: 4,838; M_PCE: 6,608) after integrating the GEX and ATAC-seq data and performing quality control based on RNA and ATAC features (nFeature and nCount). Unsupervised dimensionality reduction using weighted nearest neighbors (WNN) identified 18 transcriptionally distinct cell populations (Fig. 1a), which were well mixed across groups (Suppl. Fig. 1a). No substantial differences in RNA or ATAC quality metrics were observed among groups within clusters (Suppl. Fig. 1b-c), supporting the absence of technical bias in downstream analyses.

Cell-type annotation based on canonical marker genes identified multiple neuronal and glial populations (Fig. 1b). Among medium spiny neurons (MSNs), we resolved two Drd1^+^ subtypes—D1MSNa (*Reln*^+^) and D1MSNb (*Eya2*^+^); three types of Drd2^+^ MSN: D2MSNa (*Adamts6*^+^), D2MSNb (*Itga11*^+^), and D2MSNc (*Slc5a7*^+^); as well as a *Drd3*^+^ MSN population (D3MSN). Additional cell types included astrocytes (*Aqp4*), oligodendrocytes (OLs) (*Mog*), oligodendrocyte progenitor cells (OPCs) *(Pdgfra*), microglia (MG) (*Cx3cr1*)*,* neuron progenitor cells (NPCs) (*Nol4*). A small population of cholinergic neurons with three different subtypes was also identified based on expression of the α-7 nicotinic acetylcholine receptor (Fig.1b).

Integration of chromatin accessibility with gene expression revealed concordance between accessible regulatory regions and marker gene expression across cell types (Fig. 1b-c). Analysis of cell-type composition showed no significant differences between groups following PCE (Fig. 1d). MSNs comprised 54% of all cells and over >80% of neurons (F_Ctl: 80.7%, F_PCE: 89.2%, M_Ctl: 82.3%, and M_PCE: 77.5%), consistent with prior reports of NAc cellular composition^21^ (Fig. 1e).

We next identified differentially expressed genes (DEGs) between PCE and control rats across major cell-types (p_adj < 0.05 and |log2FoldChange| >0.35). PCE induced a greater number of DEGs in males than females across cell types (Fig. 1f). Within MSN subtypes (D1MSNa, D2MSNa, and D1MSNb), many DEGs were cell-type specific (**Suppl. Table 1**). For example, 102 DEGs were unique to male D2MSNa, compared with 59 in male D1MSNa and 34 in female D1MSNa, highlighting cell type- and sex-specific transcriptional responses to PCE (Fig. 1g). Few DEGs were shared between males and females within the same cell type, indicating pronounced sex-dependent effects. Conversely, a subset of DEGs was shared across multiple cell types (Fig. 1g), suggesting that PCE also perturbs certain common molecular pathways.

Chromatin accessibility changes were more modest overall, with comparable numbers of differential peaks between sexes across most cell types. An exception was D1MSN, where males exhibited a greater number of differential peaks (Fig. 1h). Notably, MSNs displayed more extensive chromatin remodeling than in other cell types, with differential peaks enriched in promoter regions (Fig. 1i, **Suppl. Table 2**), suggesting that PCE preferentially affect transcriptional regulation at gene initiation sites.

### PCE suppresses expression of genes involved in mitochondrial oxidative phosphorylation

**Fig. 2.**
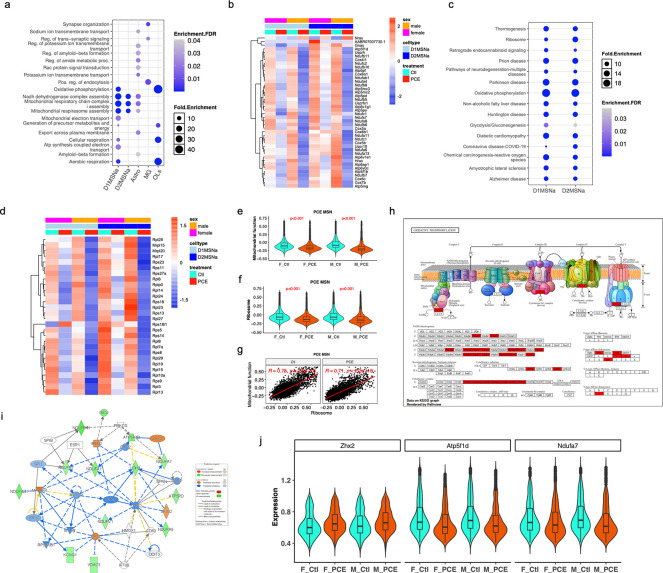
PCE induces mitochondrial dysfunction (**a**) Dot plot showing the top enriched Gene Ontology biological processes (GO: BP) based on DEGs (combined male and female) induced by PCE across five major cell types. (**b**) Heatmap displaying the expression of D1MSNa and D2MSNa DEGs enriched in GO: BP categories. (**c**) Dot plot illustrating common enriched KEGG pathways in D1MSNa and D2MSNa DEGs. (**d**) Heatmap showing the expression of D1MSNa and D2MSN DEGs enriched in ribosomal assembly pathways. (**e, f**) Violin plots showing mitochondrial function scores (**e**), and ribosome module scores (**f**) in MSNs. (**g**) Pearson correlations between ribosome module scores and mitochondrial function scores in male MSNs, shown as a representative analysis. MSNs include combined D1MSNa and D2MSNa populations. (**h**) Schematic of mitochondrial oxidative phosphorylation (OXPHOS) with PCE-associated DEGs highlighted in red. (**i**) Ingenuity Pathway Analysis (IPA) network of OXPHOS enriched from combined male and female PCE DEGs. (**j**) Violin plots showing normalized expression levels of *Zhx2*, *Atp5f1d*, and *Ndufa7* at the single-nucleus level. For clarity, only D1MSNa and D2MSNa populations were shown, as they represent the predominant D1- and D2- MSN subtypes, respectively.

Common DEGs induced by PCE in D1 and D2 MSNs were predominately associated with mitochondrial electron transport and ATP production (Fig. 2a). These genes were consistently downregulated in MSNs from both male and female offspring, with a greater magnitude of reduction observed in males (Fig. 2b). This coordinated downregulation is indicative of impaired oxidative phosphorylation (OXPHOS) and is consistent with mitochondrial dysfunction previously reported in PCE models^18, 20^.

KEGG (Kyoto Encyclopedia of Genes and Genomes) enrichment analysis of DEGs from D1 and D2 MSNs further identified ribosome-related pathways as significantly affected by PCE (Fig. 2c). Correspondingly, ribosomal gene expression was broadly reduced, suggesting impaired translation capacity in NAc MSNs (Fig. 2d). Gene module scoring based on mitochondrial or ribosomal pathway genes revealed coordinated decreases in both mitochondrial and ribosomal functional signatures (Fig. 2e-f). Notably, mitochondrial and ribosomal module scores were positively correlated (Fig. 2g), supporting a functional link between mitochondrial activity and protein translation which was disrupted by PCE. These results suggest that suppression of ribosomal function may contribute to, or occur in parallel with, mitochondrial dysfunction in PCE-exposed neurons.

Both mitochondrial and ribosomal deficits were more pronounced in male MSNs compared to females (Suppl. Fig. 2a, b), indicating sex-dependent vulnerability. In males, additional DEGs were enriched in synaptic vesicle cycle and retrograde endocannabinoid signaling pathways (Suppl. Fig. 2e). This heightened sensitivity may be related to increased expression of the cannabinoid receptor CB1 in males (Suppl. Fig. 2f), potentially amplifying THC-mediated effects.

Beyond MSNs, similar reductions in mitochondrial and ribosomal gene module scores were observed in astrocytes (Suppl. Fig. 2g, h), although the correlation between these modules was weaker than in MSNs (Suppl. Fig. 2i, g), suggesting cell type–specific coupling between metabolic and translational processes.

At the pathway level, downregulated mitochondrial genes were primarily associated with key OXPHOS components, including NADH oxidation (Complex I), cytochrome c redox reaction (Complex III/IV), and ATP synthesis (Complex V) (Fig. 2h). Upstream regulator analysis by IPA identified *Zhx2*, a Zinc-finger and homeoboxes 2 transcription factor previously implicated in metabolic regulations of mitochondrial function^22^, as a potential key mediator of PCE-induced effects on energy production (Fig. 2i). *Zhx2* expression was increased in MSNs following PCE (log2FC=0.69) and was associated with reduced expression of its predicted target genes, including *ATP5f1d* and *Ndufa7* (Fig. 2j), supporting a model in which PCE disrupts OXPHOS through regulation of nuclear-encoded mitochondrial genes.

### PCE alters chromatin accessibility at genes involved in mitochondrial function and protein homeostasis

**Fig. 3.**
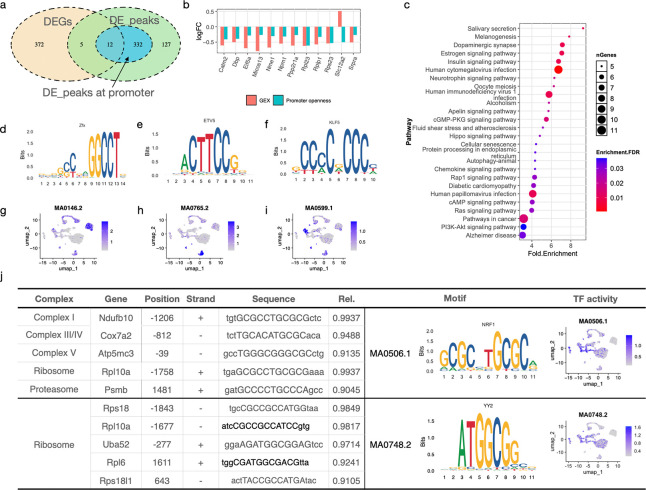
PCE alters the chromatin accessibility of mitochondrial and ribosomal genes (**a**) Venn diagram showing the overlapped genes between PCE-induced unique DEGs and genes associated with differential chromatin accessibility peaks in D1 and D2 MSNs. (**b**) The Log2 fold change (log_2_FC) of gene expression and promoter accessibility for the overlapped genes identified in (**a**). (**c**) KEGG pathway enrichment analysis of genes with differential peaks located in their promoter regions. (**d-f**) TF motifs enriched from MSN DEGs. (**g-i**) Predicted motif activity scores for MA0146.2 in oligodendrocytes and astrocytes (**g**), MA0765.2 in microglia (**h**), and MA0599.1 in neuron progenitor cells (**i**). (**j**) Summary of predicted NRF1 and YY2 biding sites identified among DEGs involved in mitochondrial, ribosomal and proteasomal activities. The relative score (Rel Score) reflects the similarity between DNA sequence patterns and reference position-specific scoring matrices (PSSMs) for known motifs; values closer 1 indicate a higher likelihood of true motif occurrence.

To investigate epigenetic mechanisms underlying PCE-induced transcriptional changes, we integrated gene expression with ATAC-seq data from male NAc cells, where PCE effects were most pronounced. In MSNs, we identified 389 DEGs and 476 genes associated with differential chromatin accessibility peaks (DPs); however, only17 DEGs overlapped with genes harboring DPs, including 12 with promoter-associated changes (TSS±2k bp) (Fig. 3a). Among these genes, chromatin accessibility was strongly correlated with transcriptional output: 11 exhibited concordant decreases in promoter accessibility and gene expression, whereas *Slc12a2* showed reduced accessibility but increased expression (Fig. 3b).

KEGG enrichment analysis of genes with promoter DPs identified signaling pathways involved in cellular metabolism and growth, including insulin, apelin, Hippo, and PI3K-Akt signaling (Fig. 3c). These pathways are known regulators of energy production and protein homeostasis, indicating that PCE may influence mitochondrial function indirectly through upstream signaling networks. Consistent with this, promoter accessibility changes were observed in key regulatory genes within these pathways, including *Pik3r2*, *Map2k1*, *Gsk3b*, *Ppp2r2d* (**Suppl. Table 3**), suggesting that PCE affects transcriptional regulation via chromatin remodeling of the upstream signaling nodes.

To further characterize regulatory mechanisms, we performed transcriptional factor (TF) motif enrichment analysis on promoter-associated DPs using Signac FindMotifs function and JASPAR2020 database. After stringent filtering (FDR<0.01 and fold-enrichment > 3), seven enriched TF motifs were identified (Suppl. Fig. 3a-c). ChromVAR analysis revealed cell type–specific motif activity patterns. For examples, motifs associated with ZFX (MA0146.2) were enriched in astrocytes and oligodendrocytes, ETV5 (MA0765.2) in microglia, and KFL5 (MA0599.1) in neuron progenitors (Fig. 3d-i), indicating distinct regulatory programs across cell types. Notably, NRF1 (MA0506.1) motif showed high predicted activity in neuronal populations and was enriched among genes associated with mitochondrial function (Fig. 3j). Using TFinder^23^ sequence analysis further demonstrated that multiple PCE-regulated mitochondrial genes including Ndufb10 (Complex I), Cox7a2 (Complex IV), and Atp5mc3 (Complex V) contain high-confident NRF binding sites. Given the established role of NRF1 in regulating nuclear-encoded mitochondrial genes^24–27^, these findings suggest that altered chromatin accessibility at NRF1 target sites may contribute to impaired mitochondrial gene expression following PCE (**Suppl. Table** 2, Fig. 3j). In addition, NRF1 binding motifs were also identified in genes involved in translation machinery (Fig. 3j, **Suppl. Table 3**), supporting a potential link between mitochondrial regulation and protein synthesis. Consistent with this, protomer regions of many ribosomal genes contained high-scoring motifs for YY2 and SP family TFs (Fig. 3g, **Suppl. Table 3**), which have been implicated in ribosomal gene regulation^28^. Together, these results suggest that PCE may coordinately affect mitochondrial function and protein homeostasis through chromatin-mediated regulation of both mitochondrial and ribosomal gene networks.

Interestingly, more than half of high-confidence DP associated sequences matched KLF5 binding motif (MA0599.1). KLF5 is known to regulate cell proliferation and differentiation and to maintain stemness of certain stem cells^29^, and high-affinity binding sites were identified in promoters of genes such as *Elk4* and *Ppp2r2d* (Suppl. Fig. 3c). ELK4 acts as a transcription factor, and PPP2r2d is part of the protein phosphatase 2 (PP2A) family. Both were known to regulate cell cycle and growth^30, 31^(Suppl. Fig. 3a), suggesting KLF5 as a master regulator in regulating MSN cell fate in the NAc. More broadly, many promoter regions contained overlapping motifs for multiple TFs (Suppl. Fig. 3b) within proximity (**Suppl. Table 4**), suggesting combinatorial regulation of gene expression in response to PCE. Furthermore, almost all seven TFs with DPs were involved in regulating cell fate, cell proliferation, or mitochondrial biogenesis, like NRF1 (Suppl. Fig. 3c), suggesting that PCE exerts a profound regulation on neuronal development and maturation in NAc.

Collectively, these findings indicate that PCE induces selective changes in chromatin accessibility at regulatory regions controlling mitochondrial function, protein synthesis, and cellular signaling, providing a potential epigenetic basis for the observed transcriptional and metabolic dysfunction.

### Acute THC challenge exacerbates mitochondrial dysfunction in PCE offspring

**Fig. 4.**
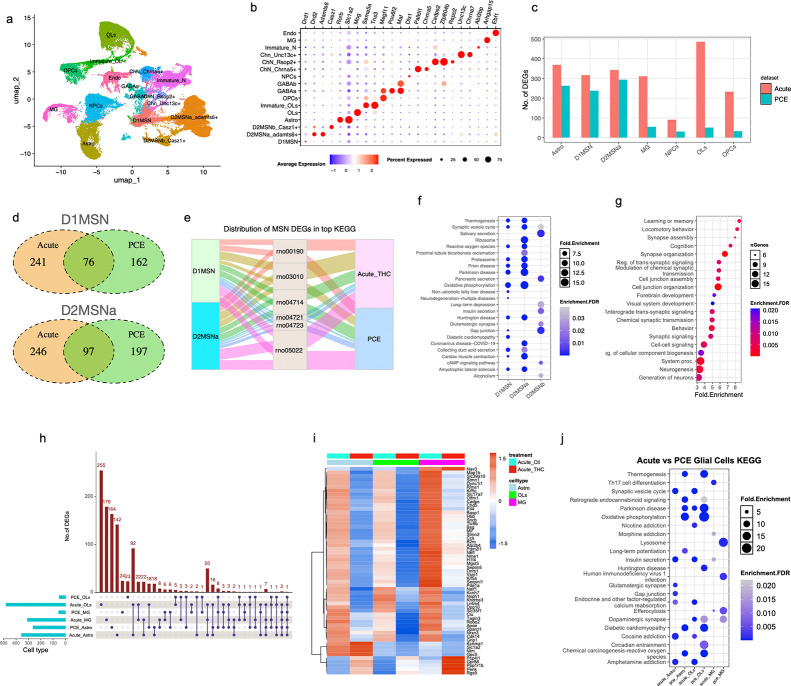
Acute THC challenge exacerbates mitochondrial dysfunction in male PCE offspring nucleus accumbens (**a**) Unsupervised clustering of single nuclei isolated from the NAc region after acute THC exposure. Each point represents a single nucleus, colored by cell cluster identity. (**b**) Dot plot showing expression of canonical marker genes used to annotate major cell types in the NAc. Dot size indicates the fraction of cells expressing each marker, and color intensity represents average expression level. (**c**) Bar plot showing the number of DEGs in major cell types after PCE alone or acute THC challenge in PCE offspring. Cell types include D1- and D2-medium spiny neurons (D1MSN, D2MSNa, D2MSNb), astro, OLs, MG, and other non-neuronal populations. (**d**) Venn diagrams depicting overlapping DEGs between PCE alone and acute THC challenge in D1MSN and D2MSNa. (**e**) Alluvial plot illustrating the distribution of D1MSNa and D2MSNa DEGs across the top KEGG pathways enriched following PCE alone or acute THC exposure. Pathways shown: oxidative phosphorylation (rno00190); ribosome (rno03010); thermogenesis (rno04714); synaptic vesicle cycle (rno04721); retrograde endocannabinoid signaling (rno04723); neurodegeneration (rno05022). (**f**) Dot plot depicting KEGG pathways enriched from acute THC challenge-induced DEGs in D1 and D2 MSNs. (**g**) Gene Ontology Biological Process (GO: BP) terms enriched from D2MSNb DEGs identified uniquely after acute THC exposure (not present with PCE alone). (**h**) UpSet plot illustrating the number of shared and unique DEGs across major non-neuron cells (astro, OLs, MG) under PCE and acute THC treatments. Horizontal bars show total DEGs per cell type/condition, and vertical bars indicate intersection sizes. (**i**) Heatmap showing expression levels of shared DEGs identified across astro, OLs, and MG in male NAc following either PCE or acute THC treatment. Rows represent genes/pathways, columns represent cell type/treatment combinations. Color scale indicates enrichment FDR. (**j**) Comparison of KEGG pathways affected by PCE alone versus acute THC exposure in astro, OLs, and MG.

Given prior evidence that adolescent THC exposure worsens PCE-associated phenotypes with male offspring exhibiting heightened vulnerability^32^, we then examined its impact on NAc transcriptional profiles specifically in male offspring. Male PCE offspring were subject to a single dosage of acute THC challenge (2.5 mg/kg, s.c.) at P24, and NAc tissue was collected four hours later for snRNA-seq analysis. After quality control and normalization, 44,215 nuclei from control and 26,228 nuclei from THC-treated rats were resolved into 18 unique clusters (Fig. 4a), with similar cell type composition and quality metrics across conditions (Fig. 4b, Suppl. Fig. 4a-c).

In contrast to PCE, acute THC challenge increased the number of DEGs across major cell types, including D1MSN, MG, OLs, and OPCs (Fig. 4c, **Suppl. Table 5**). Approximately one-third of the PCE-induced DEGs in D1MSN and D2MSNa remained differentially expressed after acute THC challenge (Fig. 4d), with similar KEGG pathway enrichment profiles (Fig. 4e), indicating reinforcement of PCE-induced transcriptional programs. he majority of MSNs, particularly D1MSNs and D2-MSNa, exhibited highly concordant responses to acute THC challenge in terms of KEGG enrichment (Fig. 4f). Notably, mitochondrial energy production pathways—especially oxidative phosphorylation and thermogenesis, were the most prominently enriched, suggesting that acute THC challenge exacerbated PCE-induced mitochondrial dysfunction. In addition, pathways associated with neurodegeneration diseases, including prion diseases, Parkinson diseases, Huntington diseases, Alzheimer diseases, and amyotrophic lateral sclerosis, were also enriched in MSNs after acute THC administration (Fig. 4f). In contrast, DEGs from D2MSNb neurons following acute THC exposure were preferentially enriched in neuronal functions-related pathways, such as synaptic vesicle cycle, gap junction, and long-term depression (Fig. 4f). These pathways were not observed in the PCE condition alone, indicating that acute THC challenge exerts a stronger subtype-specific effects on MSNs. Consistent with this, GO biological process enrichment of D2MSNb-specific DEGs revealed significant associations with learning, memory, cognition, and behavior (Fig. 4g), aligning with the established role of NAc in reward circuitry^12, 33^. Additional enriched processes included synaptic signaling and cell junction organization, and neurogenesis and brain development. Together, these findings demonstrate that a single acute THC exposure in PCE animals not only amplifies pre-existing transcriptional dysregulation but also induces distinct functional alterations in NAc MSNs.

In glial cells, acute THC exposure markedly increased the number of DEGs (Fig. 4c), the majority of which were absent in PCE alone (Fig. 4h). A substantial proportion of DEGs were shared across glial cell types: 61 common genes were identified among astrocytes (Astro), oligodendrocytes (OLs), and microglia (MG), and 160 were shared between Astro and OLs after acute THC treatment (Fig. 4h). This pattern suggests heightened sensitivity of glial cells to acute THC treatment. Across these cell types, the most shared DEGs were downregulated (Fig. 4i) and enriched in the synaptic vesicle cycle pathways (Suppl. Fig. 5a).

Notably, the expression of *Ppp1r1b* was elevated in the glial cells following acute THC treatment (Fig. 4i). In neurons, *Ppp1r1b* encodes a regulatory subunit of protein phosphatase 1 (PP1) that plays a critical role in modulating synaptic plasticity and neuronal signaling^34^. Its upregulation in glia may represent a compensatory response to impaired synaptic vesicle cycling, although its functional role in glial–neuronal interactions remains to be elucidated. Astro and OLs exhibited enrichment of similar KEGG pathways, including synaptic vesicle cycle, calcium signaling, and cocaine- and amphetamine-related pathways (Fig. 4j). In contrast, MGs displayed a distinct response profile: acute THC-induced upregulated genes were enriched in immune-related pathways such as Th17 differentiation, B cell signaling, and efferocytosis of the inflammation process, indicating an activation of inflammatory processes (Suppl. Fig. 5b-d).

Together, these results demonstrate that acute THC exposure amplifies PCE-induced transcriptional dysregulation, particularly in mitochondrial and synaptic pathways, while also eliciting additional cell type–specific responses across neuronal and glial populations.

### THC induces mitochondrial dysfunction which impairs MSN development and maturation

Mitochondrial function is a critical determinant of neuronal development and maturation^35^ providing both the energy and biosynthetic intermediates required for neurite outgrowth, synaptic formation, and neurotransmission^17, 36^. Our transcriptomic and epigenetic analyses indicated that PCE induces mitochondrial dysfunction. We next investigated whether this impairment affected the development and functional maturation of NAc MSNs.

To this end, we performed Pearson correlation analyses between mitochondrial activity and neuronal function. Mitochondrial function was quantified as a gene module derived from PCE-induced DEGs enriched for mitochondrial respiration pathways, including electron transport, OXPHOS, and ATP synthesis. Neuronal function was similarly defined using DEGs associated with axon growth, synapse organization, synaptic signaling.

**Fig. 5.**
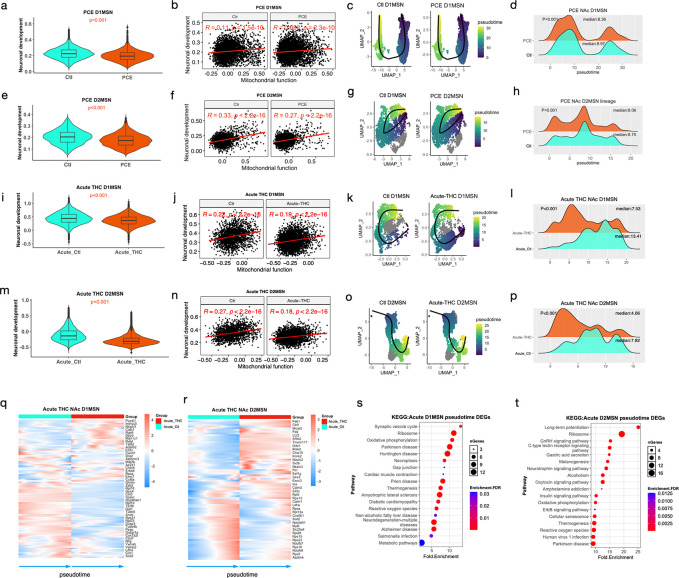
PCE-induced mitochondrial dysfunction is positively associated with neuronal development and maturation Violin plots showing gene expression module scores for neuronal development in PCE D1MSNs (**a**), PCE D2MSNs (**e**), acute THC D1MSNs (**i**), and acute THC D2MSNs (**m**). Box plots indicate the median, 25^th^ percentile, and 75^th^ percentiles. P values were calculated using the Wilcox rank-sum test. (**b, f, j, n**) Pearson correlations between mitochondrial function scores and neuronal development scores in PCE D1MSNs (**b**), PCE D2MSNs (**f**), acute THC D1MSNs (**j**), and acute THC D2MSNs (**n**). Correlation coefficient (R) and p values are shown. (**c, g, k, o**) UMAPs showing inferred trajectories of PCE D1MSNs (**c**), PCE D2MSNs (**g**), acute THC D1MSNs (**k**), and acute THC D2MSNs (**o**). The cells are colored by pseudotime and trajectories (black lines) were inferred using Slingshot. (**d, h, i, p**) Density plots showing the distributions of cells across pseudotime in PCE D1MSNs (**d**), PCE D2MSNs (**h**), acute THC D1MSNs (**i)**, and acute THC D2MSNs (**p**). Median pseudotime values and p values (Wilcox rank-sum test) are indicated. (**q, r**) Heatmaps showing dynamic expression of DEGs induced by acute THC exposure across pseudotime in D1MSNs (**q**) and D2MSNs (**r**). Gene expression trends were fitted using *associationTest* and smoothed using *predictSmooth* function, respectively, from the *tradeSeq* package. (s, t) KEGG pathway enrichment analysis of dynamically regulated acute THC challenge-induced DEGs along the MSN developmental trajectory in D1MSNs (**s**) and D2MSNs (**t**).

At P24, both D1 and D2 MSNs in PCE rats exhibited reduced neuronal development and maturation (Fig. 5a, e). Neuronal function score was positively correlated with mitochondrial respiration activity in both D1 (Fig. 5b) and D2 MSNs (Fig. 5f), suggesting that impaired mitochondrial function is associated with delayed MSN maturation, potentially due to limited availability of ATP and metabolic substrates. Acute THC administration further exacerbated the reduction in MSN developmental scores (Fig. 5i, m), which was also associated with decreased mitochondrial activity. Consistently, mitochondrial respiration and MSN development showed a moderate but significant positive correlation following acute THC challenge (Fig. 5j, n).

To characterize the temporal dynamics of THC-induced transcriptional changes, we performed pseudotime and trajectory analysis of MSN populations. PCE did not alter MSN subcluster composition (e.g., D1 MSNs; Suppl. Fig. 6a), or lineage trajectories (Fig. 5c, g, k, o). However, pseudotime analysis revealed an increased proportion of MSNs in earlier developmental states in PCE rats (Fig. 5d, h), indicating delayed maturation. This delay was further exacerbated by an acute THC challenge on P24 PCE offspring (Fig. 5i, p).

Because lineage trajectories were preserved across PCE and acute THC challenge (Fig. 5c, g, k, o), we hypothesized that THC impairs neuronal maturation primarily through transcriptional dysregulation rather than fate specification. To assess cell-type specificity, we randomly selected 2,000 non-neuronal cells from PCE and acute THC challenge offspring and determined gene module scores and correlations between mitochondrial function and neuronal development. In contrast to MSNs, non-neuronal cells showed no differences in the neuronal development scores and no correlation with mitochondrial functions (Suppl. Fig. 6b-e), supporting a neuron-specific effect.

We next examined the expression dynamics along MSN pseudotime. Genes essential for mitochondrial function (e.g., *Cox6b1*, *Cox7a2l*, *Ndufb7)*, and ribosomal components (e.g., *Rpl28* and *Rpl5)* were activated at later developmental stages but exhibited reduced expression following acute THC challenge (Fig. 5q, r). These findings suggest that both diminished energy production and impaired translation capacity contribute to delayed MSN maturation.

Additionally, genes associated with synaptic activity (*Vamp2* and *Ap2s1)*, and long-term potentiation (e.g., *Calm1* and *Calm2)* were enriched among acute THC-responsive DEGs (Fig. 5s, t) and displayed dynamic expression changes along pseudotime (Fig. 5q, r). This is consistent with the prior evidence linking mitochondrial dysfunction to synaptic impairment and neurodegeneration^36^.

## Discussion

Prenatal cannabis exposure (PCE) has been associated with an increased risk of a wide range of neurological development problems^32, 37, 38^, however, the mechanism by which PCE affects neuronal development and maturation remain poorly defined. Δ^9^-tetrahydrocannabinol (THC), the primary psychoactive component of cannabis, has been shown to impair mitochondrial energy metabolism by inhibiting oxygen consumption, reducing ATP production, and disrupting mitochondrial membrane potential across multiple cell types^39–41^. However, those observations were largely derived from cytological and pharmacological studies. Here, using single-nucleus transcriptomic and epigenomic approaches, we demonstrated that PCE suppresses gene programs, including components of electron transport chain Complex I, III, and IV, and ATP synthase, consistent with reduced mitochondrial respiratory capacity.

Notably, mitochondrial dysfunction was strongly correlated with reduced expression of ribosomal genes (Fig. 2g, j), suggesting PCE impairs not only transcriptional but also translational capacity required to sustain mitochondrial function. This interpretation is consistent with evidence that mitochondrial dysfunction, characterized by increased reactive oxygen species (ROS) formation, altered Krebs cycle metabolism, and reduced ATP production, is tightly coupled to transcriptional regulation and cell fate determination^42, 43^. Upstream regulator analysis further identified ZHX2 as a potential negative regulator of mitochondrial function in PCE offspring, consistent with prior reports that ZHX2 suppresses oxidative phosphorylation by inhibiting transcription of electron transport chain genes and destabilizing PGC-1α^22^.

Sex-specific effects were also observed, with male offspring exhibited a more pronounced reduction in nucleus-encoded mitochondrial gene expression. Male-specific DEGs were enriched in the retrograde endocannabinoid signaling pathways (Suppl. Fig. 2d), consistent with evidence that the cannabinoid 1 (CB1) receptor is present at the membrane of neuronal mitochondria (mtCB1), where its activation by exogenous cannabinoids reduces Complex I activity and mitochondrial respiration in neurons^44^ and skeletal muscles^45^. These findings raise the possibility that sex differences in mitochondrial vulnerability may reflect differential expression or activation of mtCB1in male versus female MSNs (Suppl. Fig. 2g).

Mitochondrial dynamics and localization are essential for neurite outgrowth, synaptogenesis, and overall neuronal function^17, 36, 46^. Mitochondria must be trafficked, docked, and maintained at synaptic sites, where they not only provide ATP but also act as scaffolds for the local translation of synaptic machinery assembly^47^. Consistent with this, PCE-induced mitochondrial dysfunction was positively correlated with reduced expression of genes required for MSN maturation and synaptic signaling in the NAc (Fig. 5b, f, j, n, s, t). Motif enrichment analysis revealed significant enrichment of NRF1-binding motifs (MA0506.1) in promoters of PCE-response genes (Fig. 3g). NRF1 is a key regulator of nuclear-encoded mitochondrial genes required for respiratory complex assembly^25, 27^. In line with this, NRF1 activity was predicted to be high in MSNs atP24 (Fig. 3j), and PCE reduced chromatin accessibility at NRF1-binding sites, coinciding with downregulation of mitochondrial genes.

In parallel, motif MA0748.2, recognized by YY2, was highly active in MSNs (Fig. 3j). PCE reduced the expression of ribosomal genes whose promoters are enriched for this motif (Fig. 3j), consistent with prior evidence that ribosomal genes are major YY2 targets^28^. Together, these findings support that coordinated disruption of NRF- and YY2-mediated transcriptional programs links mitochondrial dysfunction with impaired translational capacity and cellular homeostasis.

**Fig. 6.**
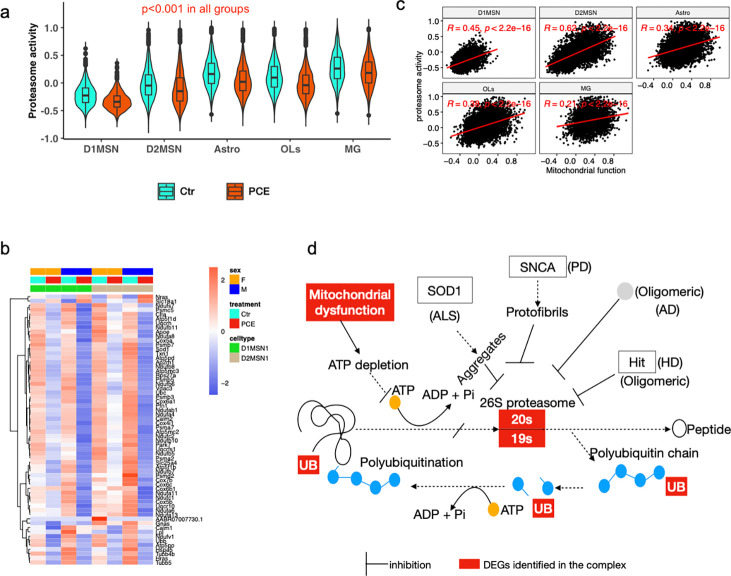
Mitochondrial dysfunction is associated with impaired protein homeostasis and neurodegenerative pathways (**a**) Violin plots showing gene expression module scores for proteasome activity across five major cell types. (**b**) Heatmap showing the expression of genes involved in protein homeostasis in D1 and D2 MSNs. Normalized snRNA-seq counts were used, and values were scaled by row. (**c**) Pearson correlations between mitochondrial function scores and proteasome activity across five major cell types. (**d**) Schematic illustrating the relationships among mitochondrial dysfunction, proteasome activity, protein homeostasis, and neurodegenerative disease pathways. Unless otherwise indicated, all analyses were performed using combined male and female data from the PCE experiment.

Importantly, PCE-induced transcriptional changes overlapped with pathways implicated in multiple neurodegenerative disease, including Parkinson’s disease (PD), Huntington’s disease (HD), amyotrophic lateral sclerosis (ALS), Alzheimer’s disease (AD), and prion disease (Fig. 2c, Fig. 4f, Suppl. Fig. 5s,t). We further observed reduced ribosome activity (Fig. 2f) and proteasome activity (Fig. 6a), accompanied by decreased expression of genes encoding these pathways (Fig. 6b). The reduction in proteasome activity was positively correlated with mitochondrial dysfunction, with the strongest correlation observed in D2 MSNs (Fig 6c), indicating that mitochondrial energy production is crucial for maintaining protein homeostasis.

For clarity, we illustrated the connections between mitochondrial dysfunction, protein degradation, and neurodegenerative diseases in Fig. 6d. In the PCE rat brains, energy production to neurons is restricted due to mitochondrial dysfunction, leading to impaired protein synthesis, reduced ribosome activity and proteasomal degradation, and accumulation of misfolded or damaged proteins which are hallmarks of neurodegeneration^48^. Beyond their role in mature neurons, mitochondria are also essential for normal neuronal function^17^, neuronal development, synaptic plasticity^49, 50^, and the regulation of development timing for cortical neurons^17^. Our findings support a model in which PCE-induced mitochondrial dysfunction disrupts MSN maturation in postnatal NAc and may increase vulnerability to neurodegenerative processes later in life through sustained impairment of protein homeostasis.

In summary, our findings identify mitochondrial dysfunction as a central mechanism linking prenatal THC exposure to impaired MSN maturation in the nucleus accumbens. Integrative transcriptomic and epigenomic analyses reveal that coordinated disruption of NRF1- and YY2-dependent genes couples with deficits in mitochondrial respiration and impaired protein homeostasis. These alterations delay neuronal maturation and may increase vulnerability to neurodegenerative processes. Together, this work establishes a mechanistic framework for how prenatal cannabis exposure perturbs reward circuitry development and highlights mitochondrial regulation as a potential therapeutic target.

Future studies will be required to determine whether these mitochondrial and proteostatic alterations persist into adulthood and contribute causally to behavioral phenotypes associated with PCE.

## Methods

### Animal care and prenatal THC exposure

All animal experiments involving animal care and sample preparation were approved by the Institutional Animal Care and Use Committee of Loma Linda University. We have complied with all relevant ethical regulations for animal use and the details in animal care were previously described^51^. Pregnant Sprague-Dawley (SD) rats, 3 months old, were purchased from Charles River Laboratories and were randomly divided into two groups: PCE group and control group. THC (20 mg/ml in ethanol) was obtained from the National Institute on Drug Abuse Drug Supply Program, and the THC solution was prepared as described^32^. From gestation day 5 (GD5) to GD20, the animals were administered either THC (2 mg/kg) or vehicle subcutaneously (s.c.) once each day. Offspring was weaned at about postnatal day 21 and maintained without any further manipulation in standard conditions. On P24, eight male rats were divided into two groups and subjected to a one-time acute THC challenge or vehicle (Ctl) s.c. injection (2.5mg/kg). The remaining animals were used for PCE sample collection. We selected P24 as it corresponds to human pre-adolescence which is a sensitive stage for acute THC exposure, and this specific dose has been shown to not induce the typical behavioral responses observed in the cannabinoid tetrad assay, nor does it lead to cannabinoid tolerance^32^. In the acute exposure experiment, samples were collected 4 hours after the s.c. injection.

### Brain tissue dissection

Rat pups on P24 were euthanized by decapitation under deep isoflurane anesthesia. The nuclear accumbens (NAc) regions were isolated on dry ice from the brain slice using a tissue punch (2mm OD). Punctured NAc and mPFC samples were pooled separately from four male or female rats from 3 dams in each group for nuclei isolation and four 10x Multiome single nuclei captures were conducted (M_Ctl, M_PCE, F_Ctl, and F_PCE). We did not use more than two rats of same sex from each litter for the same experiment to control for litter effects. Tissues from acute THC experiment were collected the same way and pooled from four animals from each group for 10× 3’ gene expression study.

### 10x snRNA-seq and Multiome (snRNA-seq and snATAC-seq) library preparation

Single nuclei isolation was performed following the 10x Genomics protocol (CG000124 Isolation of Nuclei for Single Cell RNA Sequencing) and 10,000 nuclei were targeted for each sample in both snRNA-seq and Multiome captures. snRNA-seq libraries were constructed following the established 10x Chromium Next GEM Single-cell 3’ protocol (CG000315 Rev E). For Multiome sample preparation, the resuspended nuclei were subjected to Tn5 transposition proceeding to single cell partitioning into gel beads in emulsion, barcoding. cDNA amplification. The ATAC library construction was conducted by following 10x Genomics protocols CG0001168 Rev C. Library quantification was conducted using Qubit 4.0 (Life Technologies), and quality control was assessed using a TapeStation 2200 and D1000 ScreenTape (Agilent Technologies). All sequencing libraries were prepared at the Center for Genomics at Loma Linda University (LLU). The pooled libraries were sequenced on an Illumina NextSeq 2000 (LLU Center for Genomics) with paired-end sequencing using following setting: Read 1: 28 bp, i7 index: 10 bp, i5 index: 10 bp, Read 2: 90 bp for gene expression libraries and Read 1: 50 bp, i7 index: 8 bp, i5 index: 24 bp, Read 2: 50 bp for snATAC-seq libraries.

### Reads mapping, data quality control, and dimensional reduction

Rnor 7.0 reference genome was downloaded from 10x Genomics (refdata-gex-mRatBN7–2-2024A). snRNA-seq data was mapped and counted using cellranger 8.0.1. Multiome data was mapped using cellrange-arc 2.0.2. Cells with fewer than 500 RNA features and ATAC peaks were removed. The RNA and ATAC matrices were further filtered by removing the 2% cells with the highest reads. The clustering of single nuclei based on RNA profiles was performed using the Seurat package 5.0^52^. Briefly, cell-to-gene counts were normalized, and variable genes were selected for dimension reduction by Principal Component Analysis (PCA), visualized with UMAP and clustered with the Louvain algorithm. snATAC-seq data was analyzed using Signac^53^. Specifically, ATAC common peaks matrices were binarized and normalized by Run Term Frequency Inverse Document Frequency (TF-IDF) method, followed by dimension reduction by PCA, visualization with UMAP on “lsi” reduction. To cluster single nuclei with joint modalities, snRNA-seq data and DNA cell-to-bin data were first subject to dimension reduction using “pca” or “lsi”, respectively. Then, FindMultiModalNeighbors function was used to integrate the two modalities using the first 30 components for “pca” and second to 30th components for “lsi”. Finally, cells were visualized with UMAP using Weighted Nearest Neighbor (WNN) analysis.

### Downstream data analysis

After dimension reduction, distinct clusters were defined by their projections on UMAP and the FindMarkers function was used to extract the cluster markers which were cross-referenced with known cell markers to identify cell types. Differentially expressed genes (DEGs) and differential peaks between treatment (either PCE or acute THC) and control groups were determined using FindMarkers function and significance was defined by “MAST” method with FDR<0.05 and |log2FoldChange| > 0.35. Clusters with fewer than 50 cells were excluded. Differential peaks were annotated to Rnor 7.0 genome using annotatePeak function from R package CHIPseeker^54^. MSN cells were extracted from the PCE or acute THC datasets. Following re-normalization, reclustering, the UMAP values were used for trajectory analysis using slingshot^55^. Pseudotime values for the major lineage (lineag1) were used for data presentation.

### Canonical pathway and molecular function analysis

Analyses of the gene bio-functional pathways were performed using an online analysis tool ShinyGo (v8.05, http://bioinformatics.sdstate.edu/go/). We also used the IPA (Qiagen) to identify the gene network and upstream regulators. Motif enrichment was performed with the online version of TFinder^56^.

### Statistics and reproducibility

Cell proportion was calculated by combining two subgroups (female and male) in each treatment group (PCE, acute THC challenge and control), respectively. To compare the difference, p values were calculated using Wilcox. For single nucleus RNA-seq and histone data, four animals (from two dams) were pooled in each group (female control, male control, female PCE, and male PCE). The differential expression or differential 5kb bin peak was determined using a non-parametric Wilcoxon rank-sum test as part of the Seurat package. Both p-value and adjusted p-value were reported in single nucleus sequencing data.

## Supplementary Material

Supplement 1

## Code availability

The snRNA-seq data were analyzed using Seurat (https://github.com/satijalab/seurat) and the Multiome data was analyzed using Signac (https://github.com/stuart-lab/signac). Slingshot (https://nbisweden.github.io/workshop-archive/workshop-scRNAseq/2020-01-27/labs/compiled/slingshot/slingshot.html) and Tradeseq (https://github.com/statOmics/tradeSeq) was used for trajectory analysis. No in-house tool was used.
