# Supplementary material for: Prenatal THC exposure disrupts mitochondrial respiratory gene programs and delays medium spiny neuron maturation in the nucleus accumbens
Source: bioRxiv. 2026 Apr 28:2026.04.26.720961. Preprint. [Version 1] doi: 10.64898/2026.04.26.720961 (PMC13142399; doi:10.64898/2026.04.26.720961)
Supplement: Supplementary file 1 [file media-1.pdf]

Supplementary Figures

Suppl. Fig.1

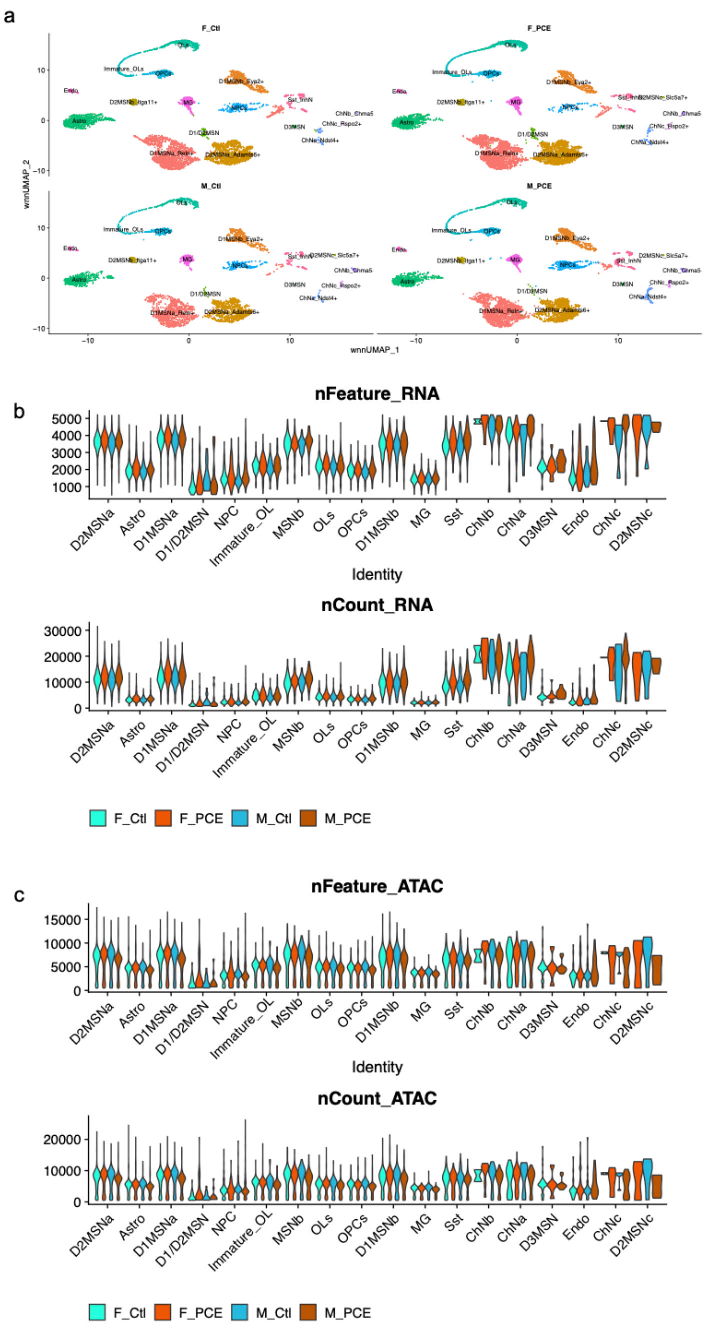

Suppl. Fig. 1. PCE dataset quality control

(a) UMAP showing the consistency in the single nuclei clustering across the four datasets. (b) Violin plots showing the RNA feature and RNA count in each cell type across the four datasets. (c) Violin plots showing the ATAC feature and count in each cell type across the four datasets.

Suppl. Fig.2

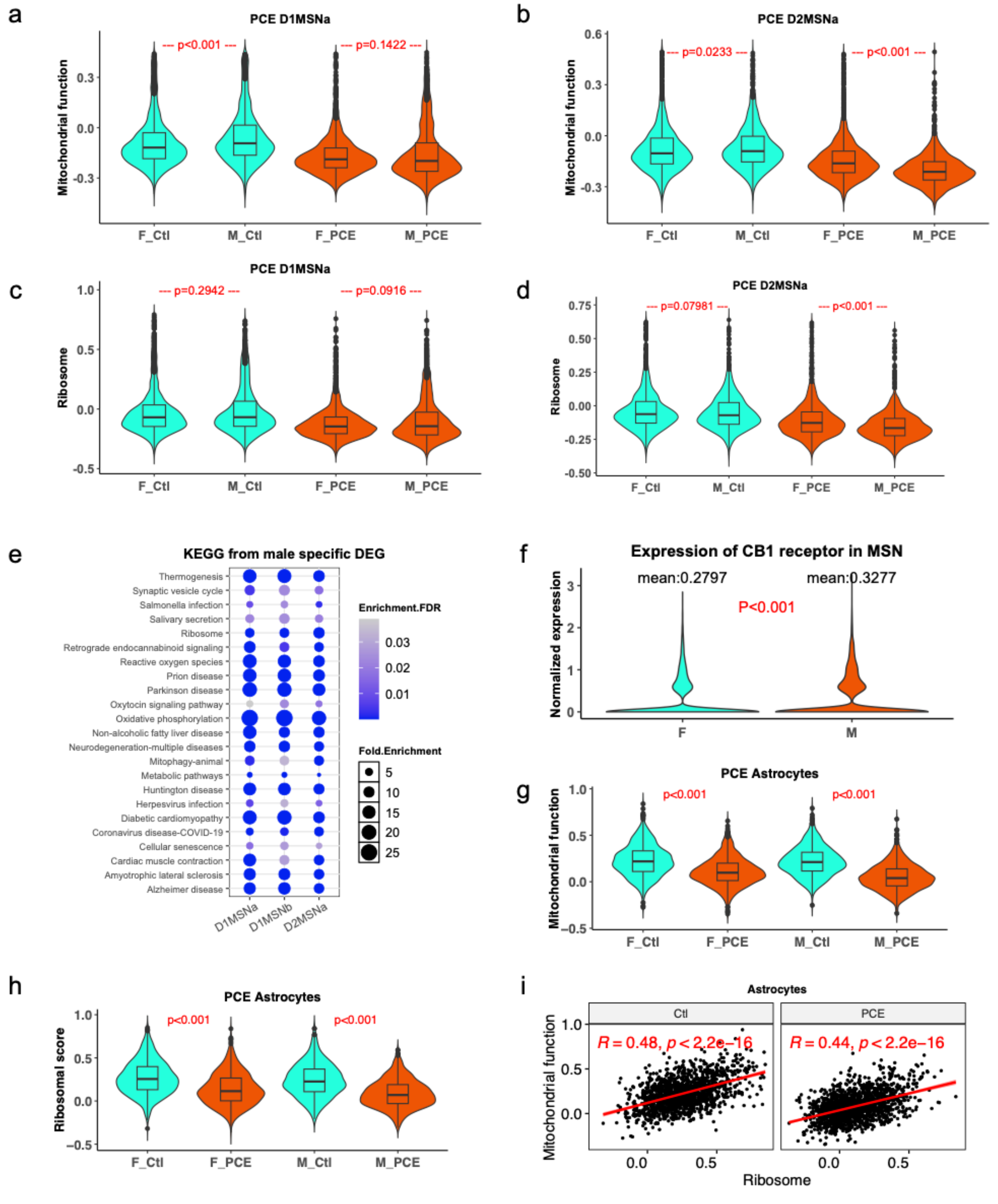

Suppl. Fig. 2. PCE inhibits mitochondrial and ribosomal activity

Violin plots showing the mitochondrial function scores in D1MSNa (a), D2MSNa (b), and ribosomal activity in D1MSNa (c), D2MSNa (d), respectively. (e) KEGG enrichment from male specific DEGs induced by PCE in D1MSNa, D1MSNb, and D2MSNa cells. (e) Violin plots showing the expression of CB1 receptor in all MSNs. (f) Violin plot showing the expression of mitochondrial CB1 receptor (mtCB1) in all MSN neurons in control animals. Violin plots showing the mitochondrial (g) and ribosomal (h) activity in astrocytes. (i) Pearson correlations between astrocyte ribosomal activity and mitochondrial function in male NAc after PCE.

Suppl. Fig. 3

a

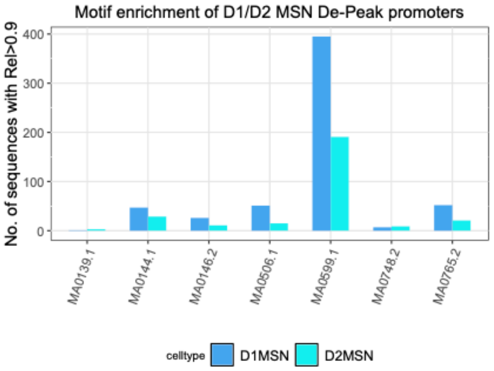

b

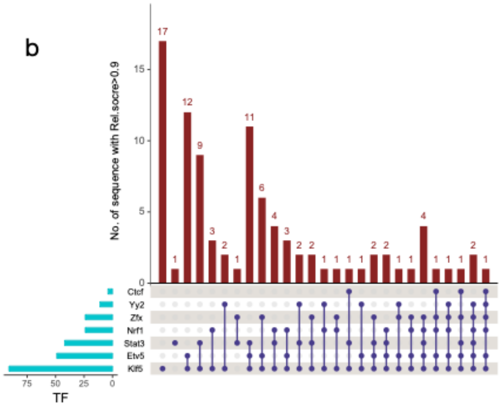

c

Motif logo	JASPRA ID	TF	TF function	Top matched sequence	Rel. score	Top matched gene
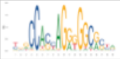	MA0139.1	Ctcf	Ctcf is involved in regulating genes that control cell fate and lineage specification during embryogenesis.	gtcCTTCCACTAGG TGGCGCTGtga	0.941	Trim35
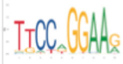	MA0144.1	Stat3	Stat3 functions as a transcription factor that regulates genes involved in cell growth, survival, differentiation, and immune responses.	ctgCTTCCTGGAAg gt	1.000	Hsp90ab1
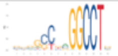	MA0146.2	Zfx	Zfx is implicated in promoting cell proliferation and survival. It can influence the cell growth and differentiation.	tcaGAGGCCTGGG CGCCcac	0.970	Zbtb7a
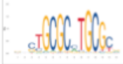	MA0506.1	Nrf1	Nrf1 is involved in the regulation of genes that promote mitochondrial biogenesis and function. It helps maintain mitochondrial health.	cacTGCGCAGGCG Cgtt	1.000	Tmem107
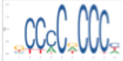	MA0599.1	Klf5	Klf5 is essential for maintaining the stemness of certain stem cells. It helps regulate pathways that keep stem cells undifferentiated and capable of self-renewal.	cctGCCCCGCCCC aga	1.000	Elk4, Hook3 Ppp2r2d
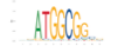	MA0748.2	Yy2	Yy2 is involved in developmental processes, influencing the differentiation and proliferation of various cell types.	aagCACCGCCATC Tctc	0.972	Mett123
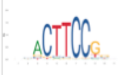	MA0765.2	Etv5	ETV5 is important for embryonic development and is involved in the differentiation of various cell types. It plays a role in the development of the nervous system, gonads.	ctgGCCGGAAGTG Ccgg	0.996	Adcy5

Suppl. Fig. 3. TF binding motifs enriched based on differential chromatin accessibility peaks induced by PCE in MSNs

a. Bar plot showing the number of DNA sequences with a Rel. score > 0.9 compared to the known TF motifs. b. UpSet plot illustrating the number of unique and common motif sequences reorganized by the seven TFs identified from. c. A table summarizing the functions of the TFs and the top potential target sequences enriched from the differential peaks induced by PCE**.** Rel. score is a measure of the similarity between a DNA sequence and a known motif sequence. The closes it is to 1, the more likely it is a true TF binding sequence. The motif enrichment was performed using the differential peaks that were annotated to a promoter (TSS±2k bp).

Suppl. Fig.4

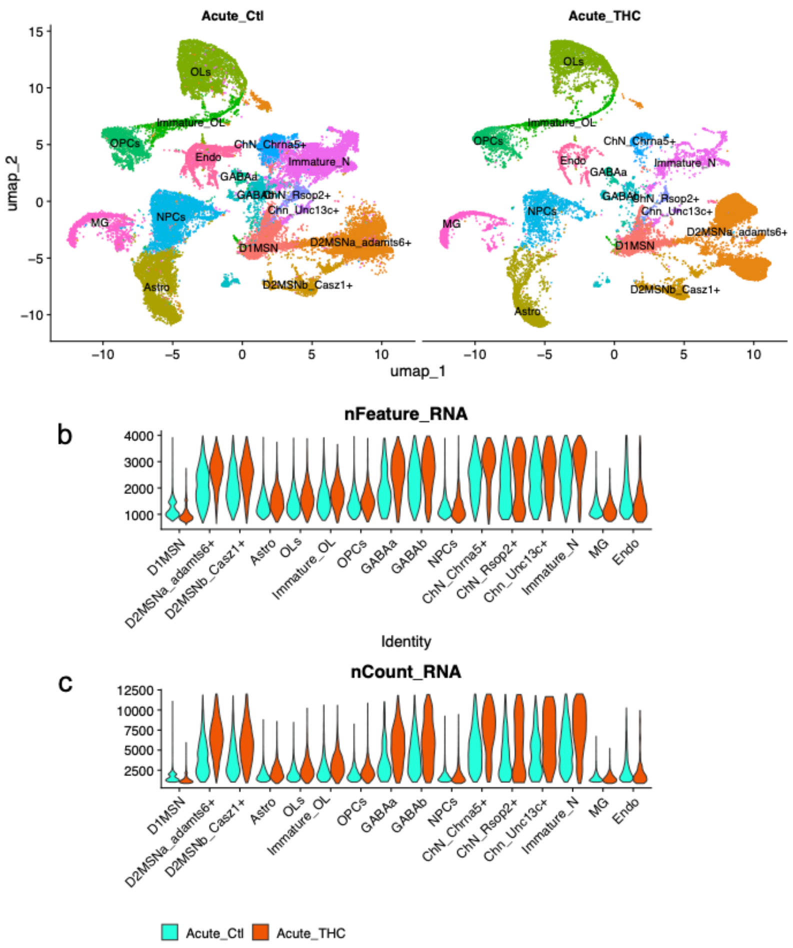

Suppl. Fig. 4. Acute THC challenge dataset quality control

(a) UMAP showing the cell types identified in NAc after acute THC exposure. Violin plots showing the distributions of RNA feature (b) and RNA count (c) across cell types in NAc, respectively.

Suppl. Fig.5

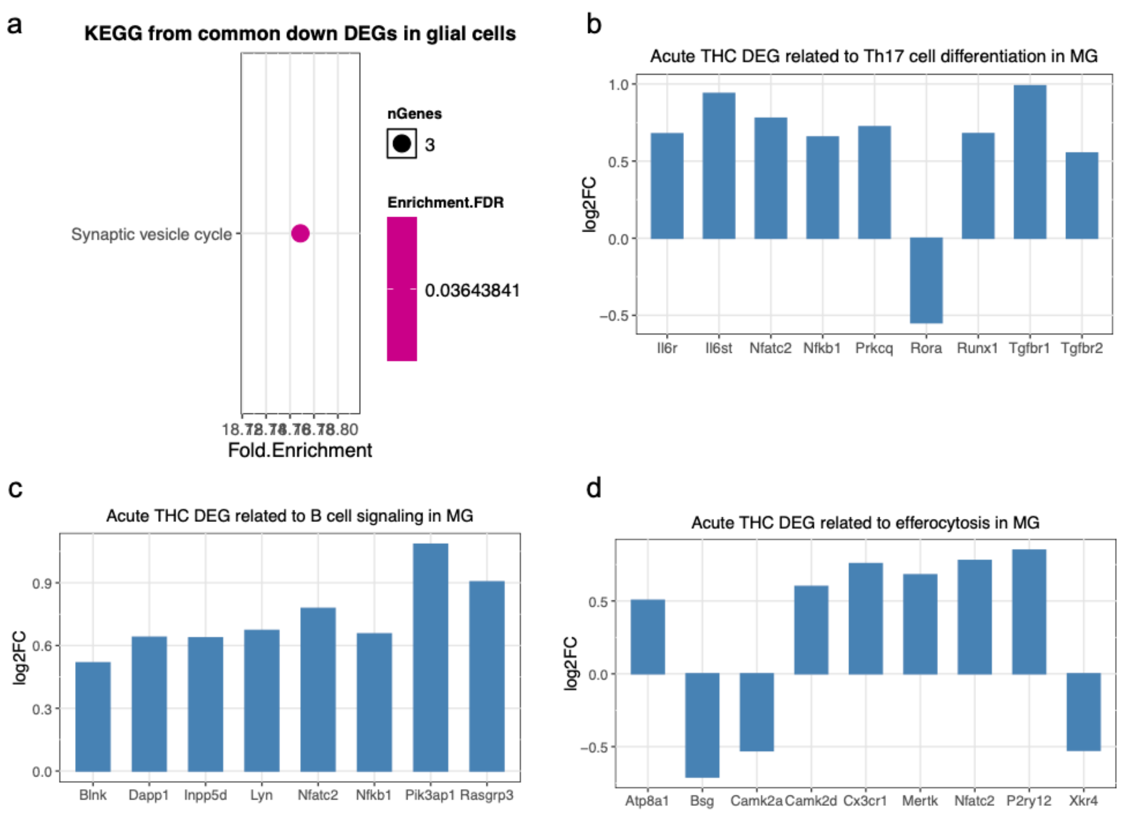

Suppl. Fig. 5. Effects of acute THC challenge on glial cells in PCE offspring

(a) KEGG pathway enriched from the common DEGs down-regulated in glial cells after acute THC exposure. Violin plots showing the log_2FC of acute THC induced DEGs in microglia related to inflammation responses: Th17 cell differentiation (b), B cell signaling (c), and efferocytosis (d), respectively.

Suppl. Fig.6

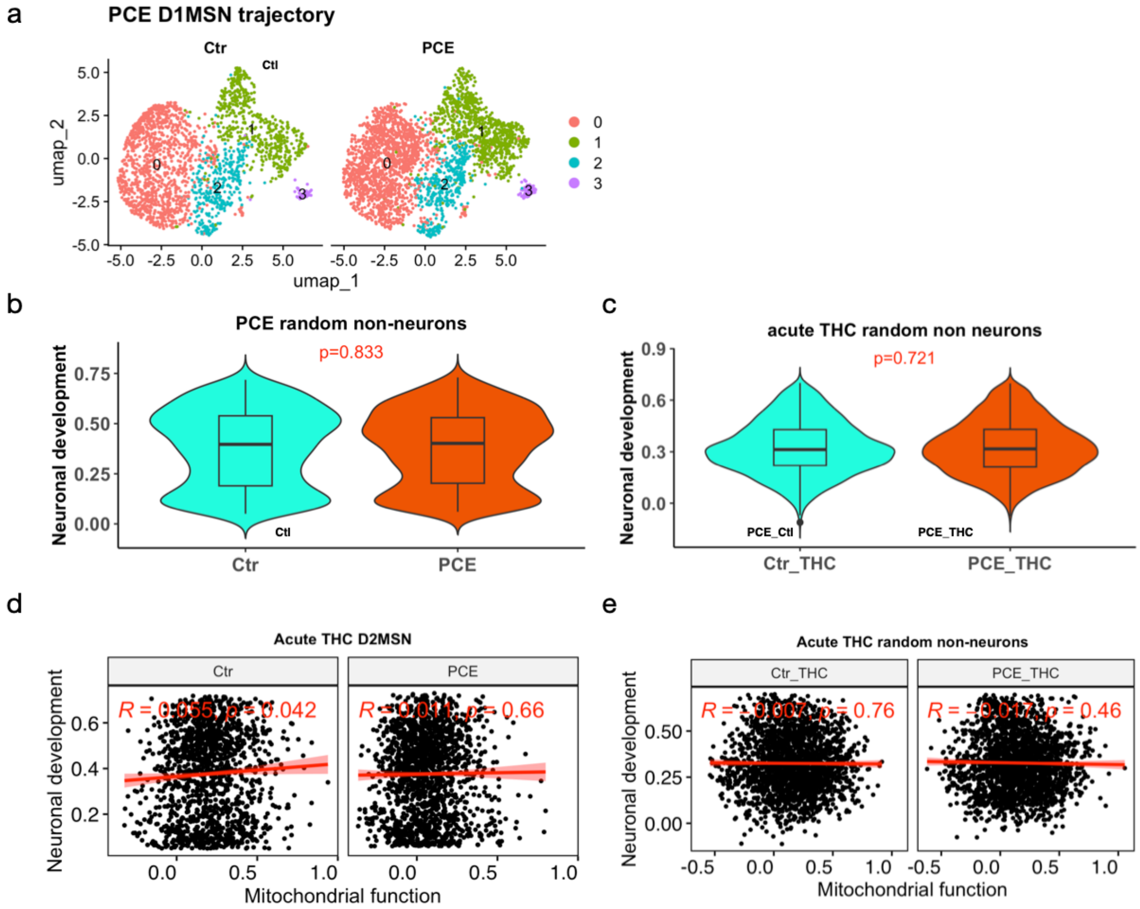

Suppl. Fig. 6. Randomization control analysis on the effects of acute THC exposure on neurodevelopment in PCE offspring

(a) UMAP showing the similar cluster pattern of D1 MSN subclusters in Ctl and PCE samples. Violin plots showing the gene module score for neurodevelopment in 2000 randomly selected non-neurons in PCE (b) and acute THC (c) dataset, respectively. Pearson correlation between the neuronal development module score and mitochondrial functions in the random non-neurons in PCE (d) and acute THC (e).
